# Supplementary material for: An InP-based vortex beam emitter with monolithically integrated laser
Source: Nat Commun. 2018 Jul 9;9:2652. doi: 10.1038/s41467-018-05170-z (PMC6037758; doi:10.1038/s41467-018-05170-z)
Supplement: Supplementary file 1 — Supplementary Information [file 41467_2018_5170_MOESM1_ESM.pdf]

Supplementary Information for
An InP-based Vortex Beam Emitter with Monolithically Integrated Laser

Zhang et al.

Supplementary Note 1: Device design

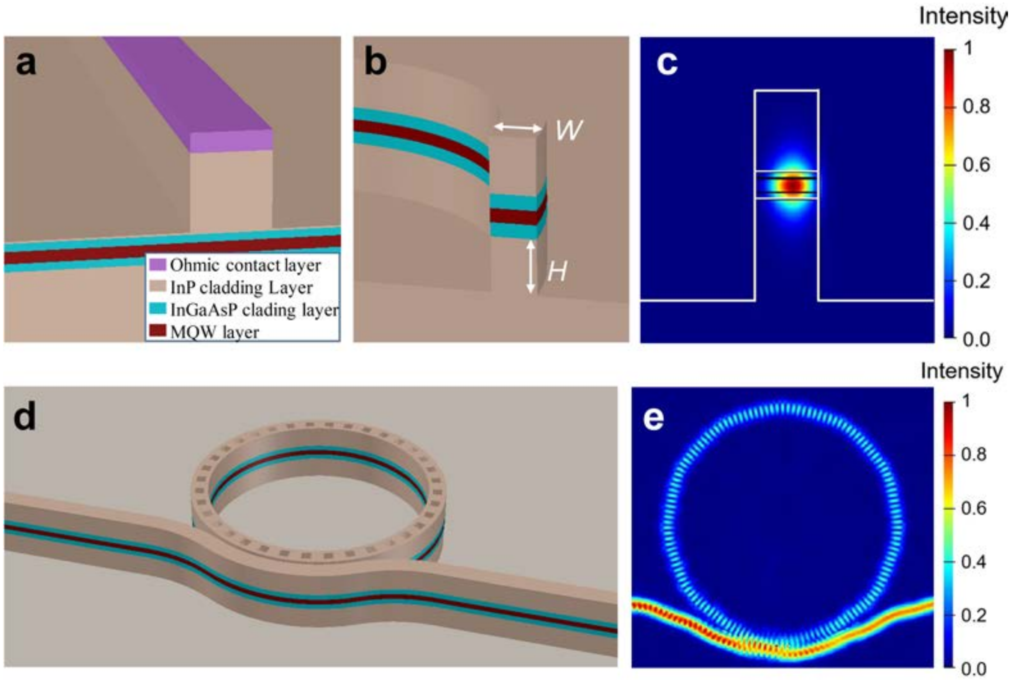

Supplementary Figure 1 | (**a**) The standard ridge waveguide and (**b**) deeply etched structure on InGaAsP/InP wafer. (**c**) Optical field of quasi–transverse electric (TE) mode in the deeply etched structure with bend radius R = 5.52 μm. (**d**) Orbital angular momentum (OAM) emitter based on deeply etched structure. (**e**) whispering-gallery-mode (WGM) in the microring.

Supplementary Figure 1a shows the standard ridge waveguide adopted for the distributed feedback (DFB) laser. The thickness of the multiple quantum well (MQW) active layer, including the top and bottom InGaAsP layers, is about 260 nm. The shallow-etched ridge waveguide is formed by removing top InP cladding layer on both side of the ridge, while the InGaAsP MQW active layer is left intact.

Though the standard ridge waveguide serves pretty well in a semiconductor laser, it suffers from unacceptably high bending radiation loss in a microring resonator. To reduce the bending loss in an InP-based microring, a deeply etched ridge waveguide structure is adopted, as illustrated in Supplementary Fig. 1b. According to our simulations, the waveguide width W of such a waveguide should be less than 1 μm to be single mode. Meanwhile, to keep optical leakage into the InP substrate at a negligible level, the distance between the ridge bottom and the InGaAsP cladding layer H should be greater than 1 μm.

Supplementary Figure 1c depicts the mode profile in a ring resonator with W = 0.8 μm, H = 1 μm, and radius R = 5.52 μm, where a small bend radius results in an asymmetric optical field distribution. Here, to reduce the simulation time and save computer memory, a microring with a radius smaller than that of the actually fabricated device is considered. It is evident that the optical mode is well confined. Simulations reveal that the deeply etched ridge waveguide exhibits a high optical confinement factor of 0.97 at 1550 nm, as compared to 0.08 for a standard ridge waveguide. As revealed in Supplementary Tab. I, thanks to the improved optical confinement of the deeply etched ridge waveguide, the bending loss can be ignored for ring resonators with a radius less than 10 μm. In addition, a smaller ring radius also helps reduce absorption loss within the resonator.

Supplementary Figure 1d illustrates the optical vortex emitter based on deeply etched ridge waveguide. As the enhanced optical confinement tends to reduce the coupling between

the microring and the bus waveguide, a pulley coupler is adopted to increase the coupling length, as illustrated in Supplementary Fig. 1d. The WGM within the ring resonator is depicted in Supplementary Fig. 1e.

Supplementary Table 1. Variation of bending loss with microring radius

Radius (μm)	5.5	10	20	40	50	100
Bending loss (dB/circle)	0.58	0.77	1.50	2.35	2.82	4.29

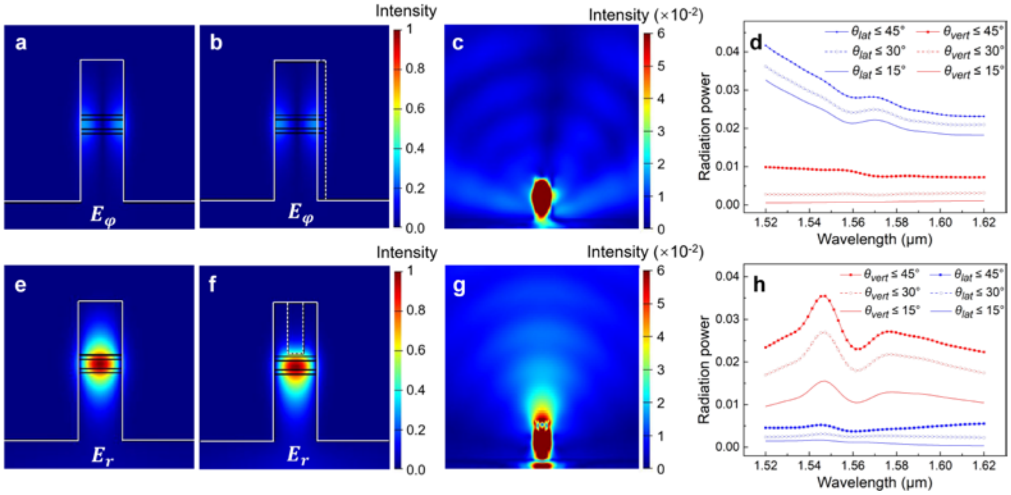

Supplementary Figure 2 | (**a**) Azimuthal electric field component E_φ and (**e**) transverse electric field component E_r for quasi-TE mode in deeply etched straight waveguide at 1550 nm. (**b**) E_φ and (**f**) E_r in waveguide with gratings at the sidewall and on the top respectively. (**c**) Lateral and (**g**) vertical radiation scattered by the grating elements located at the sidewall and on the top of the deeply etched straight waveguide respectively, normalized to the power in the waveguide. (**d**) and (**h**) are the power radiated by a 20-μm-long waveguide with sidewall and top gratings, respectively. Red and blue curves depict the radiation into the region within 45° (squares), 30° (circles), and 15° (triangles) relative to the vertical and lateral directions, respectively.

It is readily recognized that the efficiency of OAM emission critically depends on the location of the gratings. Supplementary Figs. 2a and S2e depict the azimuthal and radial components of the electric field in the deeply etched waveguide. The parameters W and H are the same as in Supplementary Fig. 1. The azimuthal component E_φ, which mainly locates at waveguide sidewall, can be scattered by grating elements positioned at the sidewall, as has been demonstrated in optical vortex emitters on silicon-on-insulator (SOI) wafer. In the deeply etched InP waveguide, however, the component E_φ at the sidewall is relatively weak as shown in Supplementary Fig. 2a. Furthermore, our simulations reveal that the sidewall gratings (depicted by the dotted line in Supplementary Fig. 2b) will radiate a considerable portion of scattered light laterally, as shown in Supplementary Fig. 2c. Supplementary Figure 2d illustrates the power of lateral (blue) and vertical (red) emission of the sidewall gratings normalized to the power in the waveguide. Total power radiated into the region within 45°, 30°, and 15° relative to the horizontal or vertical directions is calculated. It is evident that instead of forming vertically propagating OAM-carrying beams, the light scattered by the sidewall gratings is dissipated into emission in a direction close to the surface of the device. This problem is found to be especially prominent in the deeply etched waveguide. As their height increases, the gratings located at the waveguide sidewall behave

more like a quasi-one-dimensional vertical grating and the radiation beam is approximately horizontal.

On the other hand, Supplementary Fig. 2e reveals a strong radial component E_r at the centre of the deeply etched InP waveguide. If grating elements are formed on the top of the waveguide (as illustrated by the dotted line in Supplementary Fig. 2f), E_r will be scattered to form a vertical emission as demonstrated in Supplementary Figs. 2g and 2h, resulting in a radially polarized OAM-carrying beam.

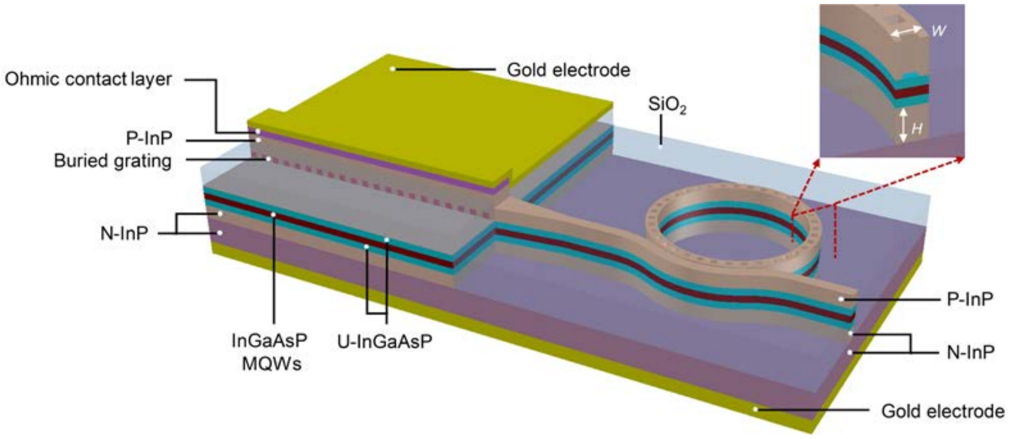

Supplementary Figure 3 | Integrated OAM laser. Inset is the cross section of the OAM emitter.

The top-grating structure and the corresponding OAM laser are illustrated in Supplementary Fig. 3. The sidewalls of grating region are retained, making it looks as if composed of "holes". The advantage of such configuration is that the waveguide with grating holes can be fabricated by a single-step dry etching process (see Supplementary Note 3: Device fabrication).

Supplementary Note 2: Simulation results. In our device, OAM-carrying beams are generated when the following angular phase-matching condition is satisfied

$$l = M - N \tag{1}$$

where l, M, and N are all integers, with l being the topological charge of the OAM state, N the number of total grating elements, and M the azimuthal resonant order of the WGM.

Supplementary Figure 4a shows the simulated transmission and emission spectra of the designed OAM emitter normalized to the power in the bus waveguide, ignoring the absorption loss and scattering due to sidewall roughness. A microring radius of R = 5.52 μm and a total grating element number of N = 72 are adopted. The parameters of the ring waveguide, W and H, are the same as in Supplementary Fig. 1, while the size of the grating hole is 0.4×0.3 μm^2, and its depth 0.85 μm. Effective coupling between the microring and the pulley coupling waveguide can be secured for a gap size of 40 nm, as confirmed by the transmission spectrum. The peaks in emission spectrum correspond to the OAM-carrying beam emitted vertically at the resonant wavelength. As the input wavelength increases from 1520 nm to 1620 nm, the WGM order M changes from 72 to 68. According to (1), the OAM order l of the emitted beam varies from 0 to −4.

Supplementary Figure 4b depicts the intensity profile of the emitted beam with l = −2, captured in the vertical direction of the OAM emitter. The pattern is concentric annular with a dark centre, a tell-tale feature of OAM-carrying beam.

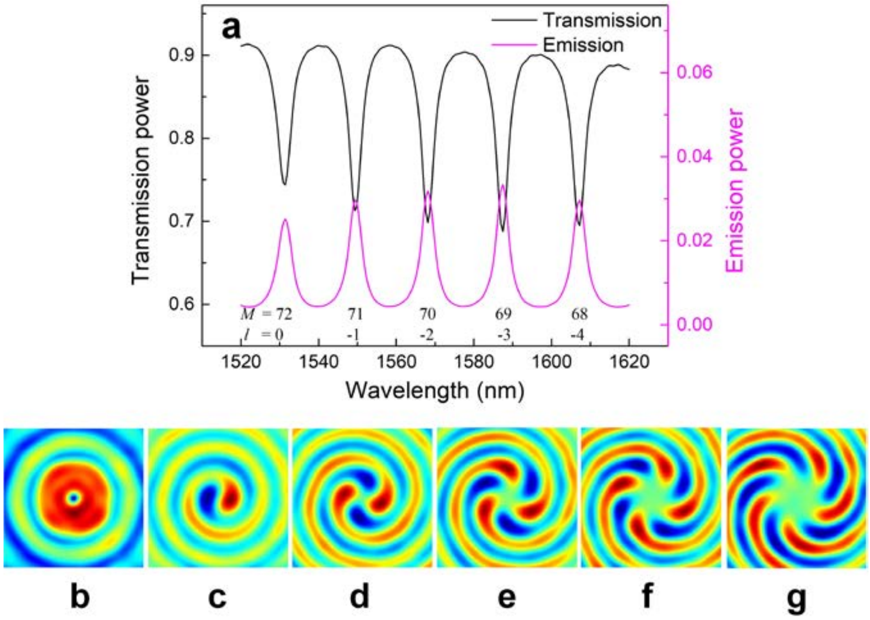

Supplementary Figure 4 | (**a**) Simulated transmission and emission spectra. (**b**) Simulated radiation pattern for l = -2. (**c-g**) Simulated helical phase patterns of the OAM-carrying beams, with $l - 1$ = -1, -2, -3, -4, -5 respectively.

The field of OAM beam radiated by gratings on the WGM resonator can be described by Jones vector as

$$\mathbf{E} = E_{c1}\exp[\mathrm{j}(l+1)\varphi]\begin{pmatrix}1\\ -\mathrm{j}\end{pmatrix} + E_{c2}\exp[\mathrm{j}(l-1)\varphi]\begin{pmatrix}1\\ \mathrm{j}\end{pmatrix}, \quad (2)$$

which is composed of a right-hand circularly polarized (RHCP) OAM beam with topological charge of $l + 1$ and a left-hand circularly polarized (LHCP) OAM beam with $l - 1$. Supplementary Figures 4c to 4g are the simulated phase patterns of the LHCP component of the emitted OAM beam, with the number of the helical arms equal to $|l - 1|$.

Supplementary Note 3: Device fabrication. Supplementary Figure 3 shows the schematic of the OAM laser formed by monolithic integration of a shallow-etched DFB laser with a deeply etched optical vortex emitter.

A two-step metal organic chemical vapor deposition (MOCVD) growth is adopted to complete the epitaxial wafer. During the first growth, the MQW layer consisting of 10 periods of 6 nm compressively strained InGaAsP well and 9 nm tensile strained InGaAsP barrier, sandwiched between two 60 nm InGaAsP cladding layers, is grown on top of an n-InP substrate. First order gratings are then formed over the DFB laser section by holographic exposure and wet-etching. During the second growth, the entire wafer is covered by 1.7-μm-thick p-InP cladding layer and 250-nm-thick p^+-InGaAs ohmic contact layer

The DFB laser is fabricated over the region with buried gratings and processed into a standard ridge waveguide structure, whereas the deeply etched optical vortex emitter is formed on the grating free region. The ohmic contact layer and part of the top InP-cladding layer over the vortex emitter region are removed, so as to reduce absorption of the vertical OAM emission. The height of the vortex emitter is 2.1 μm, with the top and bottom InP layer in the deeply etched ridge waveguide being 0.85 and 1 μm, respectively. The microring and the bus waveguide are both 0.8 μm wide, and a taper is adopted to connect the 2-μm-wide DFB laser to the bus waveguide. The fabrication procedures are summarized in Supplementary Fig. 5.

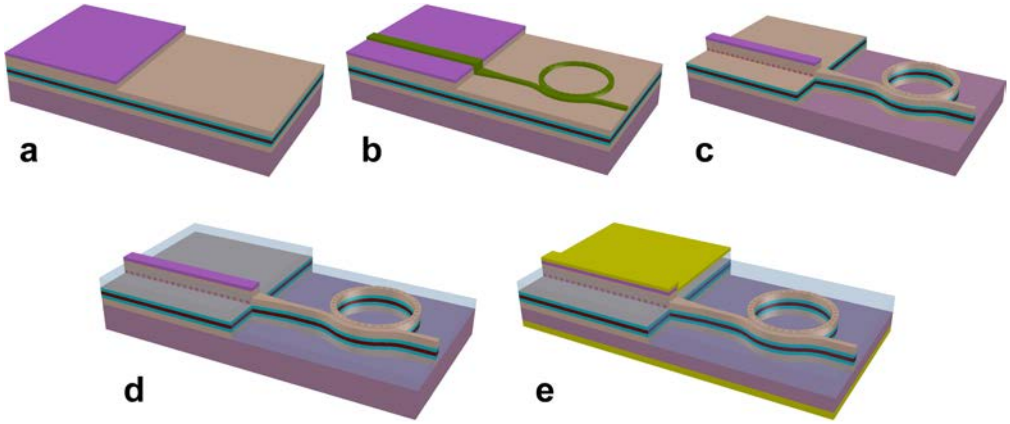

Supplementary Figure 5 | Fabrication procedures of integrated OAM laser. (**a**) Partial removal of ohmic contact layer and top InP cladding layer. (**b)** SiN_x mask formation of by electron beam lithography (EBL) and reactive ion etching (RIE). (**c**) H_2/CH_4 inductively coupled plasma (ICP) dry etching. (**d**) SiO_2 deposition. (**e**) Electrode formation.

One of the challenges in device fabrication is the formation of top gratings, which consists of a series blind holes drilled into the microring waveguide. As shown in the inset of Supplementary Fig. 3, the sidewalls of grating region are retained, making it possible to fabricate the waveguide with grating holes by a single-step dry etching process. It is well known that dry etching process exhibits lag effect, i.e., the etch rate is dependent on the mask opening. As a result, the etch rate within the grating holes is lower than in the open area. Consequently, by adjusting the size of the grating holes and tuning the dry etching conditions, the deeply etched waveguide and shallow-etched gratings can be formed by a single-step dry etching progress. This scheme makes it possible to protect the MQW layer beneath the grating region from being etched, thus ensuring a low transmission loss in the WGM resonator.

Supplementary Table 2. Control of grating hole depth

**No.**	**Hole size (nm^2)**	**ICP etching**						**Hole depth (nm)**
		RF power (W)	ICP power (W)	Pressure (mTorr)	H_2 Flux (sccm)	CH_4 Flux (sccm)	Step time (s)	
**a**	380×400	80	80	15	38	17	45	1038
**b**	200×230	80	80	15	38	17	45	562
**c**	200×260	100	0	15	38	17	60	765
**d**	200×260	100	0	15	33	22	120	667

Supplementary Table 2 and Supplementary Fig. 6 illustrate how to modify the depth of grating holes. To facilitate observation of the top-grating structure by scanning electron microscopy (SEM), it is fabricated on a straight waveguide. The height of all the waveguides is about 2 μm. ICP etching using H_2 and CH_4 mixture are carried out with a chamber pressure of 15 mTorr. During the etching process, a 20-second O_2 plasma cleaning is performed after each etching step, so as to clean the etching product covering the wafer. Thus H_2/CH_4 etching alternates with O_2 plasma cleaning during the ICP etching process.

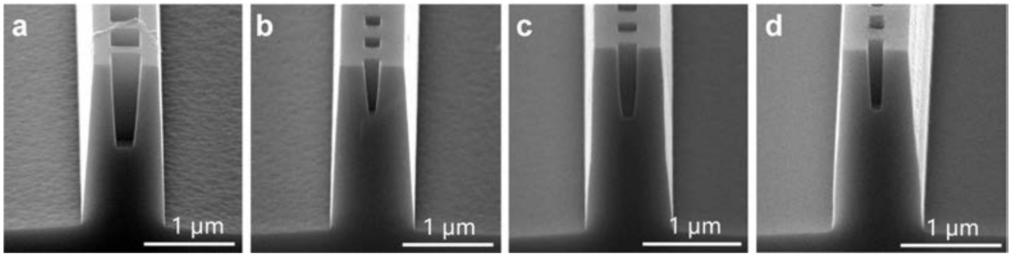

Supplementary Figure 6 | SEM image of the top-grating structure fabricated by EBL and ICP dry etching. Each figure corresponds to the condition listed in Supplementary Tab. II.

Comparing Samples a with b, it is clear that a shallow hole could be obtained by reducing the size of the mask opening. On the other hand, Samples c and d reveal the effect of ICP etching conditions. As CH_4 helps protect the etched surface, increasing CH_4 flow rate results in reduced hole depth. Supplementary Figure 6 shows that grating holes with smooth surface morphology and controllable depth can be fabricated. As a result, the MQW layer beneath the grating region can be protected by adjusting the top size of holes and ICP etching conditions according to the thickness of the top InP cladding layer.

Supplementary Note 4: Equipment and settings. Key equipment, image acquisition tools and image processing software packages are list in the Supplementary Tab. III.

Supplementary Table 3. Equipment used in device fabrication and characterization

Item	Equipment type
EBL for Figure 1 and Supplementary Figure 6	Raith eLINE Plus
SEM for imaging Figure 1	ZEISS_MERLIN
Objective lens for imaging Figures 2(c-g) and Figure 4(a)	Thorlabs, RMS40X-PF
Infrared CCD for Figures 2(c-g) and Figure 4(a)	Hamamatsu, C10633-23
Software for Figure 5	Lumerical MODE and Lumerical FDTD
Software for Supplementary Figures 1c, 1e, 2, and 4	Lumerical MODE and Lumerical FDTD
EBL for Supplementary Figure 6	Raith eLINE Plus
SEM for Supplementary Figure 6	Raith eLINE Plus

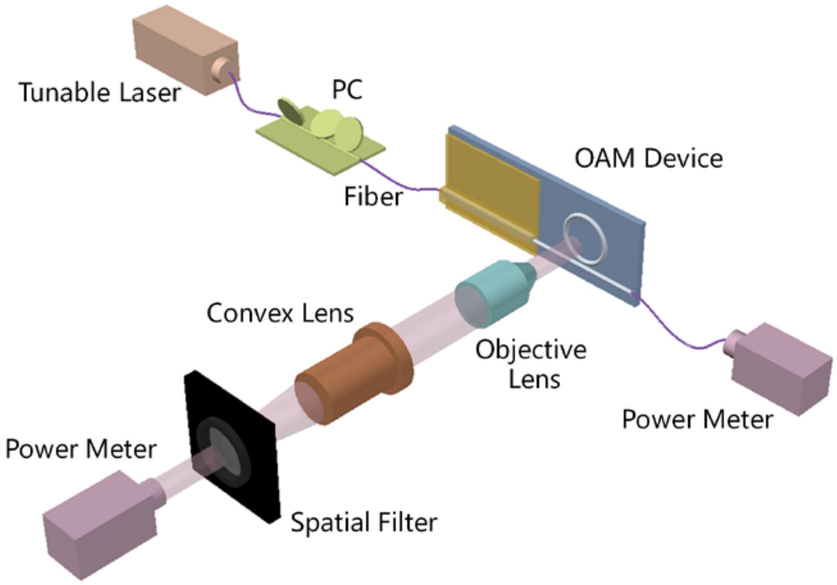

Supplementary Figure 7 | Experimental setup for transmission and emission spectra measurement. Light from a tunable laser is coupled into the DFB laser facet of the device by a lensed fibre, and a fibre-optic polarization controller (PC) is used to select TE polarized incident light. The light exiting the optical vortex emitter facet is collected by another lensed fibre and fed into a power meter. Meanwhile, light beam radiated in the vertical direction is collimated by an objective lens and recorded with a power meter. A convex lens followed by a spatial filter is used to transform it into a near field beam and suppress any stray light in the near field of the OAM-carrying beam.

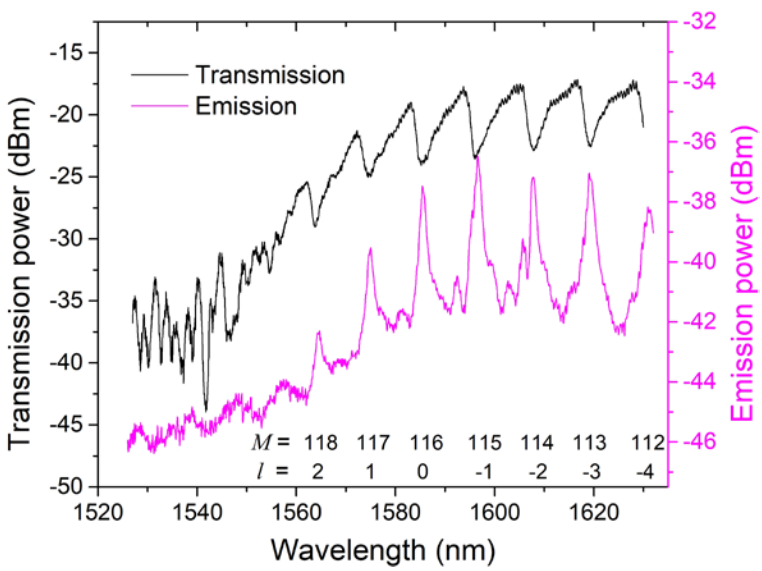

Supplementary Figure 8 | Transmission and emission spectra of the vortex emitter. The spectra were measured with no injection current into the DFB laser and the optical power of the incident light is 5 dBm. Each of the nearly equally spaced dips in the transmission spectrum corresponds to a distinctive WGM resonance (*M* value), while the corresponding peak in the emission spectrum will have a unique topological charge (*l* value), as specified by equation (1). The conspicuous drop in transmission at short wavelengths is due to absorption of the TE polarized incident light by the compressively strained InGaAsP MQW active layer[1], whereas the dip around ~1541.9 nm is due to the reflection of the DFB gratings.

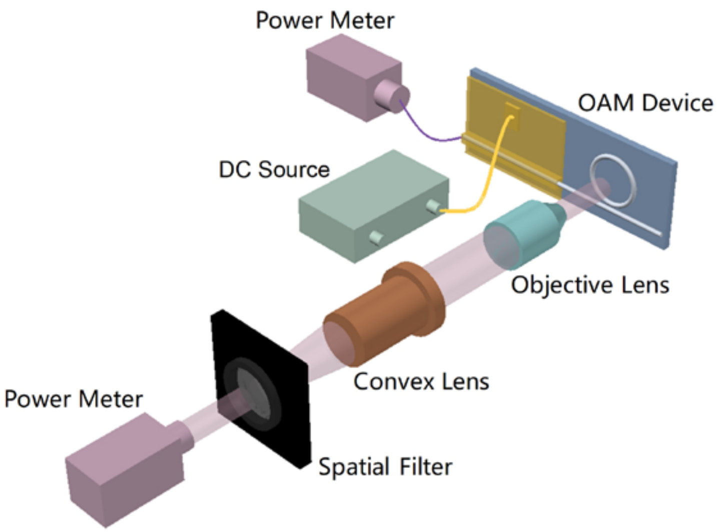

Supplementary Figure 9 | Experimental setup for characterizing L-I and polarization behavior of the OAM laser. The major deference from the system shown in Supplementary Fig. 7 is that, instead of resorting to an external laser source, the DFB laser pumped by a direct current (DC) power supply is used to excite the optical vortex emitter. L-I characteristic of the OAM laser is measured by recording the power of the DFB laser port and the vertical emission as a function of the current injected into the DFB laser. The near field pattern of the vertical emission is recorded by replacing the power meter with an infrared charge-coupled device (CCD). The polarization behavior of the emitted beam is determined by inserting a polarizer after the objective lens.

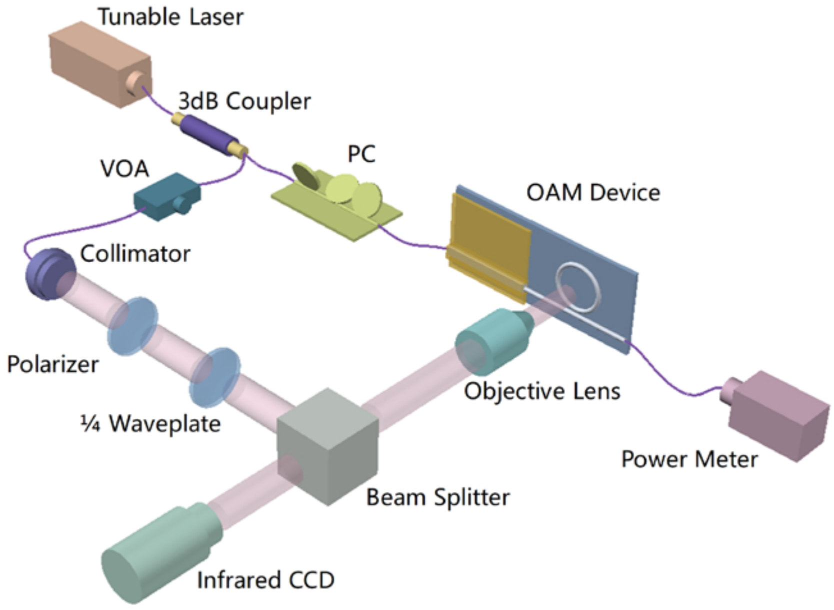

Supplementary Figure 10 | The experimental setup for investigating the phase structure of the radiated beam. The topological charge of the emitted OAM-carrying beam can also be determined by studying the helical phase structure via interference with a plan wave. In the measurement setup, the output of a external tunable laser is split into two branches by a 3dB coupler. Light from one branch is coupled into the integrated chip by a lensed fibre. Light from the other branch is used as reference beam. It is expanded into a Gaussian beam by a collimator, and transformed into LHCP or RHCP by a polarization filter (consiting of a linear polarizer and a 1/4 waveplate), with its power controlled by a variable optical attenuator (VOA). The two branches, i.e. OAM-carying beam and reference beam, are combined at a beam splitter and interference patterns is recorded by an infrared CCD.

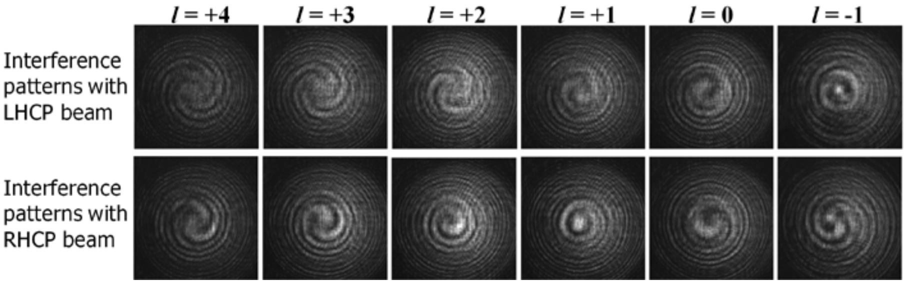

Supplementary Figure 11 | The helical phase structure of vortex beam measured by the setup in Supplementary Figure 4. OAM-carrying beam with topological charge changes from +4 to -1 interfered with LHCP and RHCP reference beams. Each pattern on the upper row has $l + 1$ spiral arms, whereas those on the lower row have $l - 1$ spiral arms. The result confirms the effectiveness of the OAM emitter in our integrated device.

Supplementary References

1. Coldren, L. A., Corzine, S. W., & Mashanovitch, M. L. *Diode lasers and photonic integrated circuits 2nd ed*. (John Wiley & Sons, Inc., Hoboken, New Jersey, 2012).
